# Pediatric patient asthma-related emergency department visits and admissions in Washington, DC, from 2001–2004, and associations with air quality, socio-economic status and age group

**DOI:** 10.1186/1476-069X-6-9

**Published:** 2007-03-21

**Authors:** Steven M Babin, Howard S Burkom, Rekha S Holtry, Nathaniel R Tabernero, Lynette D Stokes, John O Davies-Cole, Kerda DeHaan, Deitra H Lee

**Affiliations:** 1 Johns Hopkins University, Applied Physics Laboratory, 11100 Johns Hopkins Road, Laurel, MD 20723, USA; 2 Environmental Public Health Tracking Program, Bureau of Epidemiology and Health Risk Assessment, District of Columbia Department of Health, 825 North Capitol Street NE, 3rd Floor, Washington, DC 20002, USA; 3 Currently employed by the Division of Energy Employees Occupational Illness Compensation, US Department of Labor, Washington, DC 20210, USA; 4 Currently employed by the US Government Accountability Office, Washington, DC 20548, USA

## Abstract

**Background:**

The District of Columbia (DC) Department of Health, under a grant from the US Centers for Disease Control and Prevention, established an Environmental Public Health Tracking Program. As part of this program, the goals of this contextual pilot study are to quantify short-term associations between daily pediatric emergency department (ED) visits and admissions for asthma exacerbations with ozone and particulate concentrations, and broader associations with socio-economic status and age group.

**Methods:**

Data included daily counts of de-identified asthma-related pediatric ED visits for DC residents and daily ozone and particulate concentrations during 2001–2004. Daily temperature, mold, and pollen measurements were also obtained. After a cubic spline was applied to control for long-term seasonal trends in the ED data, a Poisson regression analysis was applied to the time series of daily counts for selected age groups.

**Results:**

Associations between pediatric asthma ED visits and outdoor ozone concentrations were significant and strongest for the 5–12 year-old age group, for which a 0.01-ppm increase in ozone concentration indicated a mean 3.2% increase in daily ED visits and a mean 8.3% increase in daily ED admissions. However, the 1–4 yr old age group had the highest rate of asthma-related ED visits. For 1–17 yr olds, the rates of both asthma-related ED visits and admissions increased logarithmically with the percentage of children living below the poverty threshold, slowing when this percentage exceeded 30%.

**Conclusion:**

Significant associations were found between ozone concentrations and asthma-related ED visits, especially for 5–12 year olds. The result that the most significant ozone associations were not seen in the age group (1–4 yrs) with the highest rate of asthma-related ED visits may be related to the clinical difficulty in accurately diagnosing asthma among this age group. We observed real increases in relative risk of asthma ED visits for children living in higher poverty zip codes versus other zip codes, as well as similar logarithmic relationships for visits and admissions, which implies ED over-utilization may not be a factor. These results could suggest designs for future epidemiological studies that include more information on individual exposures and other risk factors.

## Background

Elevated concentrations of ground-level ozone and particulates have been shown to be associated with increased incidence of asthma exacerbations [1-3]. Aeroallergens, such as pollen and mold, may trigger allergy-induced asthma symptoms. Seasons for various aeroallergens differ by geography, and, in Washington, DC, the following are the pollen seasons for trees, grasses, and weeds, respectively: February through June, May through August, and July through October [4]. Also, either very low or very high ambient temperature may be associated with asthma exacerbations. Higher temperatures may be associated with asthma exacerbations because they occur when there is more sunlight, and sunlight is necessary for emissions to be converted to ground-level ozone. At the other temperature extreme, studies have suggested that very cold, dry air acts as an airway irritant, yet primarily results in exacerbations of exercise-induced asthma [5-7], which is not a part of this study.

The District of Columbia Department of Health (DC DOH), under a grant from the US Centers for Disease Control and Prevention (CDC), established an Environmental Public Health Tracking Program (EPHTP) to demonstrate possible relationships between ambient ozone and particulates and short-term asthma health outcomes, and to identify areas and populations most likely to be affected by pollution [8]. Therefore, the goals of this small pilot study were to determine the degree to which asthma exacerbations are associated with ozone and particulate concentrations in the short-term, on the order of days after presumed exposure, and to identify pediatric populations that may be at increased risk of these health effects. Asthma exacerbations were determined by daily counts of pediatric ED visits for asthma-related problems. As will be described, the spatial variations among pollutant measurements on a given day were rather small. This is consistent with DC being an urban area with little industry and whose major source of ozone and PM2.5 is the considerable amount of vehicular traffic that permeates the 159 km^2 ^land area of DC. In accordance with the EPHTP goals mentioned above, we examined other spatial features that show more significant regional variation, including residence zip code and socio-economic status of the pediatric population. Some interesting results were thereby obtained, especially with regard to socio-economic status.

## Methods

The concentrations of ozone and particulates of aerodynamic diameter 2.5 μm or less (PM2.5) were obtained from available measurements at five locations within DC from October 2001 through September 2004. Ozone measurements in units of parts per million (ppm) were made hourly at:

▪ Takoma Elementary School,

▪ River Terrace Elementary School, and

▪ Southeast (SE) end of McMillan Reservoir.

Daily PM2.5 measurements in units of μgm^-3 ^were made at:

▪ River Terrace Elementary,

▪ National Park Service Office at Haines Point, and

▪ SE end of McMillan Reservoir.

Note that two of the locations above measured both ozone and PM2.5. For the purposes of our analysis, the hourly ozone data were converted into 8-hour daily maximum ozone concentrations to conform to non-attainment criteria adopted by the US EPA (see, for example, [9,10]). While PM2.5 and ozone were each measured at three different locations within DC, the spatial variation among measurements for each day during the three-year period was found to be negligible compared to differences expected to cause asthma problems. Therefore, we used the daily mean of the available measurements among these sites to represent each day's ozone or PM2.5 concentration.

Aeroallergen data were collected at the Walter Reed Army Medical Center by the US Army Centralized Allergen Extract Laboratory. While not collected every day, these data are collected about three days a week and more frequently from spring through early fall. These data covered the months of October 2001 through September 2004, except for December 2002 when no measurements were taken. These data consisted of daily counts of grass, weed, and tree pollen and mold spores in units of grains m^-3^. Daily temperature data were obtained from the National Weather Service measurements at Reagan National Airport in DC.

For health outcomes, our data consisted only of daily counts of asthma-related pediatric emergency department (ED) visits by DC residents between October 2001 and September 2004. Asthma-related visits were defined as those ED visit records in which one of the first three of nine possible diagnosis fields listed an asthma code number. These ED record fields contain numbers based on the International Classification of Diseases Ninth Revision (commonly called ICD-9) codes used by hospital and physicians' office personnel to report billing information to insurance companies [11]. ICD-9 codes may indicate either symptoms or specific diseases, and those beginning with 493 indicate an asthma diagnosis. Based on discussions with medical personnel about ED coding practices among different DC hospitals, the criterion of an asthma code in one of the first three fields was chosen as an indication that an asthma exacerbation was a principal reason for the visit. The pediatric patients were those with an age between 1 and 17 years on the ED visit date. Patient identification was not included in the data except for residence zip code, age at time of visit, date of visit, and whether or not the patient was admitted. Data were grouped into these patient age groups: 1–4 years, 5–12 years, 13–17 years, and 1–17 years. Analysis was performed independently on each of these age groups. In addition to these data, we also obtained pediatric population by zip code and age group for DC according to the 2000 US Census. This US census data also provided for each zip code the percent of the child population living in households with income considered to be below the US poverty level.

**Figure 1:**
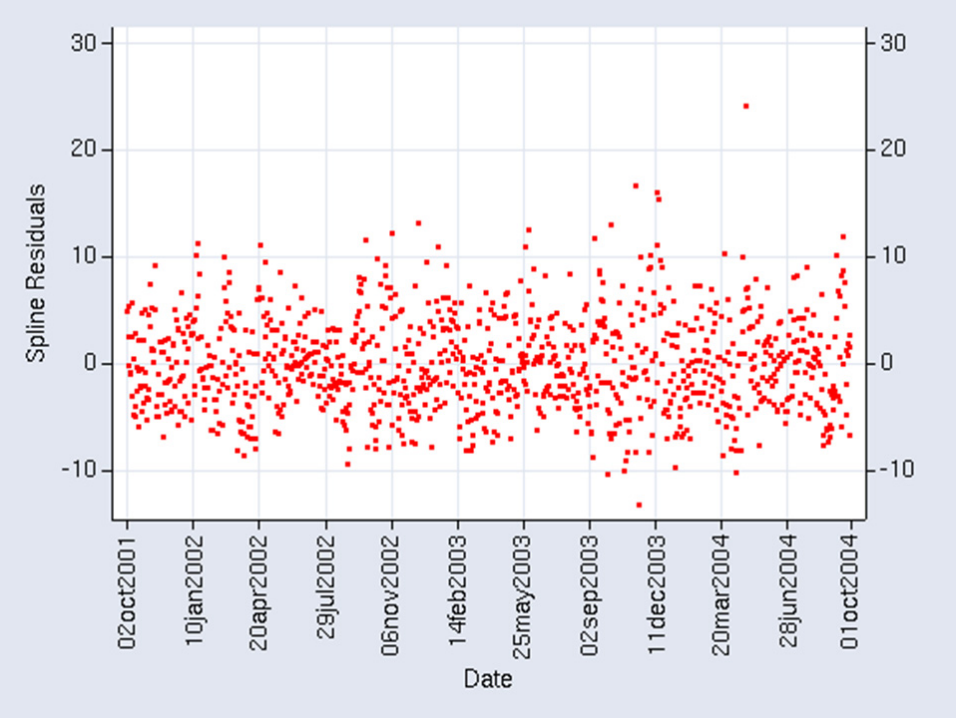
**Daily ED Visit Residuals**. Daily time series plot of the residuals of daily pediatric asthma ED visits for 1–17 yr olds (observed minus the spline predicted).

We found that there were generally two annual peaks in the daily ED visits for pediatric asthma exacerbations in DC: the highest peak in September-November and a secondary peak in March-May. Because annual peaks in ozone and PM2.5 tend to occur in the summer months, this discrepancy results from strong seasonal effects on exacerbations that are associated with other factors [12]. For example, several studies [13-15] have noted increased asthma morbidity triggered by respiratory viral infections (e.g., colds) that occur during late summer and early autumn. While we had no data on respiratory infections, their known seasonal trending was a motivator for the selection of knots for the spline to be described subsequently. We had no data on indoor and behavioral factors related to asthma exacerbations. No day-of-week effects could be identified in the asthma-related ED daily count data. Because a focus of the study was on short-term associations of air pollutants (i.e., days) and asthma ED visits and admissions, it was necessary to control for the above stronger long-term influences that were not part of the study. Many investigators have used a natural cubic spline function to represent the strong seasonal signals as a control for confounders in order to observe short-term asthma associations with pollutants [16,17]. Once the long-term trends are removed, the short-term associations become apparent. Our spline curve was derived from a best fit to the time series of daily asthma-related ED visits for DC children of ages 1–17 yrs over a three-year period. This spline had twelve knots to represent the frequency of variation of strong seasonal changes over the study period. Because there were 3 years of data and 4 seasons per year, 12 knots appeared to be a reasonable choice. These knots were not evenly spaced, but were selected to coincide with large long-term changes in the daily ED visits. Once these long-term trends were removed, the residual data (Fig 1) were examined for short-term (on the order of days) associations with ozone and PM2.5. Note in Fig. 1 that, while changes in variance remain, the long-term trends have effectively been removed.

While there were a number of choices for an analysis model, our goal was to quantify short-term associations in the time series of the ED daily counts with the environmental data. Our health data covered three years and consisted of de-identified, zip-code level, daily counts of asthma-related ED visits in a pediatric population. A Poisson regression analysis assumes that counts of independent, rare events follow a Poisson distribution and models the logarithm of daily visit counts as a linear function of the explanatory variables. The Poisson regression model adjusts for data skewness by using a log transformation and is useful for distributions of nonnegative data, and data for which the variance increases as the mean. Poisson analysis therefore seemed appropriate for daily counts of asthma-related ED visits. While other regression models may also be appropriate, we chose the Poisson regression for the above reasons and the fact that it had successfully been used in other studies similar to ours (e.g., [18-20]).

Our database for analysis included date; daily ED visit and admission counts for each age group (1–4, 5–12, 13–17, and 1–17 yrs); daily 8-hr maximum ozone concentrations (based on the mean of the available measurement sites for that day); daily PM2.5 concentrations (based on the mean of the available measurement sites for that day); daily maximum temperature; daily tree, grass, and weed pollen concentrations; daily mold concentrations; month and season; and spline curve predictions. Because exercise-induced asthma diagnosis codes were virtually non-existent in our ED data and are those asthma exacerbations associated with cold temperatures, we examined only the associations with maximum daily temperature. Significant associations in this database were determined using the statistical analysis tools contained in the Stata [21] statistical software package developed by Stata Corporation of College Station, TX. Our null hypotheses were that there were no significant associations of ozone, particulates, aeroallergen, or socioeconomic status with pediatric asthma-related ED visits.

## Results

### Same-Day Effects

For ED visits, only a slight association was found with ozone effects for the 1–17 yrs age group. However, the 5–12 yrs age group showed a much stronger association (Table 1), with a 3.2% increase in visits indicated for an increase of 0.01 ppm in ozone concentration (95% Confidence Interval, or CI, = 1.4–5.0). Significant effects of tree pollen and ambient temperature were also seen for the 5–12 yrs age group. For hospital admissions from ED visits, the analysis was significant for the 1–17 yrs age group (Table 2), with a 0.01-ppm increase in ozone being associated with a 4.5% increase in asthma-related ED admissions (95% CI = 0.6–8.5). The 5–12 yrs age group showed even greater ozone impacts (Table 2), with a 0.01-ppm increase in ozone concentration being associated with an 8.3% increase in asthma-related ED admissions (95% CI = 2.6–14.4). Weed pollen and ambient temperature also showed significant associations with ED admissions for both the 5–12 yrs and 1–17 yrs age groups. Other age groups did not show significant impacts from ozone, and no significant associations of PM2.5 were seen over this three-year period.

**Table 1:** Risk Factor Associations with ED Visits for Ages 5–12 yrs

**Risk Factor Increase**	**Risk Factor**	**Mean % Change in Daily Visits per Risk Factor Increase**	**T-test p-value**
0.01 ppm	Ozone	3.2 (1.4, 5)	0.000
1 μg m^-3^	PM2.5	-0.2 (-0.6, 0.2)	0.331
1000 spores m^-3^	Mold Count	-0.2 (-1.1, 0.7)	0.707
10 grains m^-3^	Weed Pollen	-2.9 (-6.8, 1.1)	0.148
100 grains m^-3^	Tree Pollen	1.8 (0.9, 2.6)	0.000
10 grains m^-3^	Grass Pollen	1.6 (-2, 5.4)	0.378
1°F	Temperature	0.2 (0.1, 0.4)	0.007

The numbers in parenthesis represent the lower and upper 95% confidence limits for the percent change in visits.

**Table 2:** Risk Factor Associations with ED Admissions for Ages 1–17 yrs and for Ages 5–12 yrs

**Risk Factor Unit Increase**	**Risk Factor**	**Mean % Change in Daily Visits per Risk Factor Unit Increase**	**T-test p-value**
0.01 ppm	Ozone	4.5 (0.6, 8.5)	0.023
1 μg m^-3^	PM2.5	0.2 (-0.6, 1.1)	0.548
1000 spores m^-3^	Mold Count	1.1 (-0.8, 3)	0.250
10 grains m^-3^	Weed Pollen	7.7 (0.7, 15.2)	0.029
100 grains m^-3^	Tree Pollen	1.5 (-0.3, 3.3)	0.105
10 grains m^-3^	Grass Pollen	0.7 (-7.4, 9.5)	0.874
1°F	Temperature	0.5 (0.1, 0.8)	0.018
			

**Risk Factor Unit Increase**	**Risk Factor**	**Mean % Change in Daily Visits per Risk Factor Unit Increase**	**T-test p-value**

0.01 ppm	Ozone	8.3 (2.6, 14.4)	0.004
1 μg m^-3^	PM2.5	-0.4 (-1.6, 0.8)	0.479
1000 spores m^-3^	Mold Count	2 (-0.6, 4.6)	0.140
10 grains m^-3^	Weed Pollen	10 (0.5, 20.5)	0.039
100 grains m^-3^	Tree Pollen	1.8 (-0.8, 4.4)	0.173
10 grains m^-3^	Grass Pollen	1.8 (-9.4, 14.5)	0.764
1°F	Temperature	0.9 (0.4, 1.5)	0.001

The numbers in parenthesis represent the lower and upper 95% confidence limits for the percent change in admissions. The upper table is for ages 1–17 yrs and the lower table is for ages 5–12 yrs.

### Lagged Day Effects

We also looked for associations of ozone and PM2.5 concentrations with ED visits and admissions up to 4 days following anomalies in de-trended pollutant levels. These lagged effects were examined in separate models and are shown in Table 3. For ED visits, the 1–17 yrs age group showed significant associations only with grass pollen. An increase in grass pollen of 10 grains m^-3 ^was associated with a 2.6% increase in asthma-related ED visits (95% CI = 0.3–5). The 5–12 yrs age group showed significant lagged associations with ozone, tree pollen, and grass pollen. A 0.01 ppm increase in ozone was associated with a 1.9% increase (95% CI = 0.2–3.7) after 1 day, a 2.3% increase (95% CI = 0.6–4.1) after 2 days, a 2.8% increase (95% CI = 1.1–4.6) after 3 days, and a 3.3% increase (95% CI = 1.6–5.1) after 4 days, for asthma-related ED visits.

**Table 3:** Lagged Day Associations of ED Visits with Ozone and Pollen.

Visits 1–17 yrs						
	Ozone		Tree Pollen		Grass Pollen	

Lag (days)	Mean % Change in Daily Visits per Unit	T-test p-value	Mean % Change in Daily Visits per Unit	T-test p-value	Mean % Change in Daily Visits per Unit	T-test p-value

0	1 (-0.1, 2.1)	0.078	0.5 (0, 1.1)	0.068	0.7 (-1.8, 3.1)	0.602
1	0.1 (-1, 1.2)	0.879	0.4 (-0.2, 1)	0.202	1.9 (-0.4, 4.3)	0.112
2	-0.2 (-1.3, 0.9)	0.724	0.5 (-0.1, 1.1)	0.105	2.3 (-0.1, 4.7)	0.056
3	0.3 (-0.8, 1.5)	0.572	0.2 (-0.4, 0.8)	0.492	2.6 (0.3, 5)	0.030
4	0.2 (-0.9, 1.3)	0.754	0.2 (-0.4, 0.9)	0.441	-0.4 (-2.9, 2.3)	0.787
						

Visits 5–12 yrs						

	Ozone		Tree Pollen		Grass Pollen	

Lag (days)	Mean % Change in Daily Visits per Unit	T-test p-value	Mean % Change in Daily Visits per Unit	T-test p-value	Mean % Change in Daily Visits per Unit	T-test p-value

0	3.2 (1.4, 5)	0.000	1.8 (0.9, 2.6)	0.0001	1.6 (-2, 5.4)	0.378
1	1.9 (0.2, 3.7)	0.030	1.2 (0.4, 2.1)	0.005	3.5 (0.1, 7.1)	0.046
2	2.3 (0.6, 4.1)	0.009	1.5 (0.7, 2.4)	0.0002	4.3 (0.9, 7.9)	0.013
3	2.8 (1.1, 4.6)	0.002	1.1 (0.3, 2)	0.01	5.5 (2.2, 9)	0.001
4	3.3 (1.6, 5.1)	0.000	1.1 (0.2, 2)	0.016	3.1 (-0.5, 6.8)	0.088

The top and bottom tables are for the 1–17 yr old and 5–12 yr old age groups, respectively. The mean % change in daily visits in these tables is per unit 0.01 ppm increase in ozone, 100 grains m^-3 ^increase in tree pollen, and 10 grains m^-3 ^increase in grass pollen.

A 100 grains m^-3 ^increase in tree pollen was associated with a 1.2% increase (95% CI = 0.4–2.1) after 1 day, a 1.5% increase (95% CI = 0.7–2.4) after 2 days, a 1.1% increase (95% CI = 0.3–2) after 3 days, and a 1.1% increase (95% CI = 0.2–2) after 4 days, for asthma-related ED visits for 5–12 yr olds. Grass pollen also showed a progressively increasing effect when lagged from 1 to 3 days, and the association diminished for greater lags. Specifically, a 10 grains m^-3 ^increase in grass pollen was associated with a 3.5% increase (95% CI = 0.1–7.1) after 1 day, a 4.3% increase (0.9–7.9) after 2 days, a 5.5% increase (95% CI = 2.2–9) after 3 days, and a 3.1 % increase (95% CI = -0.5–6.8) after 4 days, for asthma-related ED visits.

### Covariate Interactions

We analyzed the data for interactions among ozone, PM2.5, and different types of pollen and mold spore counts. However, no significant interactions were identified, so these results are not shown. This result may be due to the small number of high pollen days in the 36 months of data.

### Socioeconomic Status

**Figure 2:**
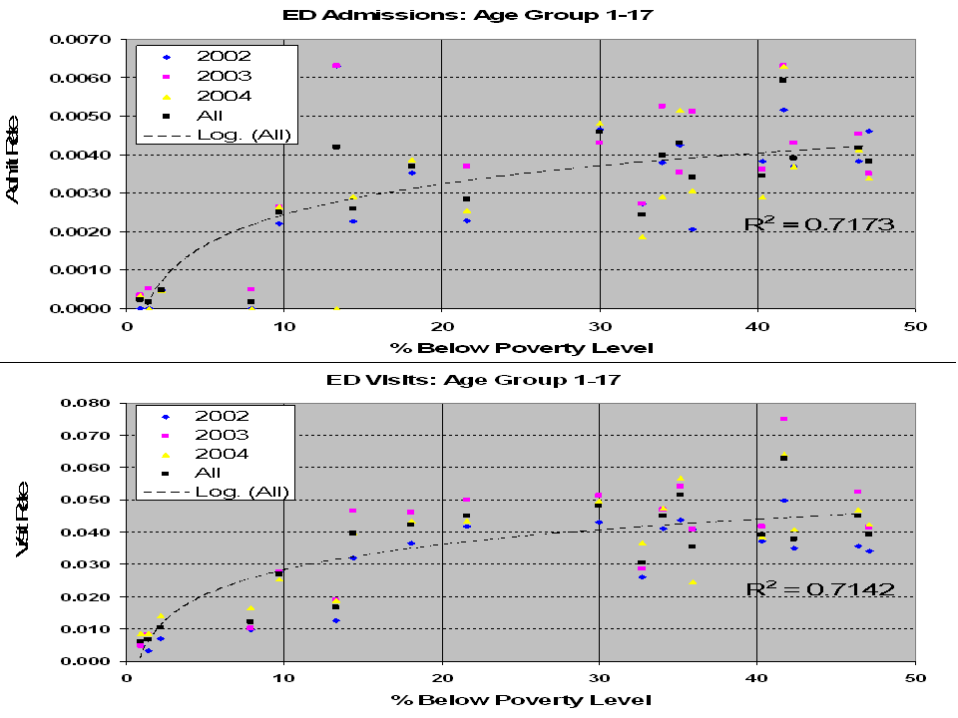
**ED Visit and Admission Rates for Ages 1–17 Yrs vs Percent below Poverty Level in Zip Code**. Upper plot shows the asthma-related ED admit rate and the lower plot shows the asthma-related ED visit rate for children ages 1–17 years versus the percent of children living below poverty levels for zip codes within DC.

As mentioned earlier, one of the goals of the EPHTP is to identify areas and populations that may be at greater risk of health effects of pollution [8]. Because the ozone and PM2.5 concentrations did not vary appreciably by location within DC, we compared the geospatial information of residence in zip codes of low socioeconomic status with other zip codes. The asthma-related ED visit rate for each zip code was determined by dividing the number of asthma-related ED visits from a zip code by the pediatric population of that zip code. When this ED visit rate was plotted against the percentage of children living in households with income below poverty level, a logarithmic relationship was observed (Fig. 2). Similar results were obtained when ED admission rate was used instead of visit rate. Note that there is a strong, nearly linear relationship as the percent below poverty rises to nearly 5%. Above 5%, the relationship becomes curvilinear. Above about 30%, the relationship becomes almost linear again but with a very slow rise. Based on our literature search, we believe this is the first time such a logarithmic relationship has been observed between asthma-related ED visit or admission rates and pediatric socio-economic status. These logarithmic correlations are very high (R^2 ^close to unity), indicating that this relationship is highly significant. Fig. 2 shows both asthma-related ED visit and admission rates increased with the percentage of the pediatric population below poverty level, suggesting that over-utilization of hospital ED for routine primary care of asthma exacerbation does not explain the positive slope of these curves. Together, the curves in Fig. 2 suggest a real increased risk of acute asthma exacerbations as the percent of children below poverty level increases.

**Figure 3:**
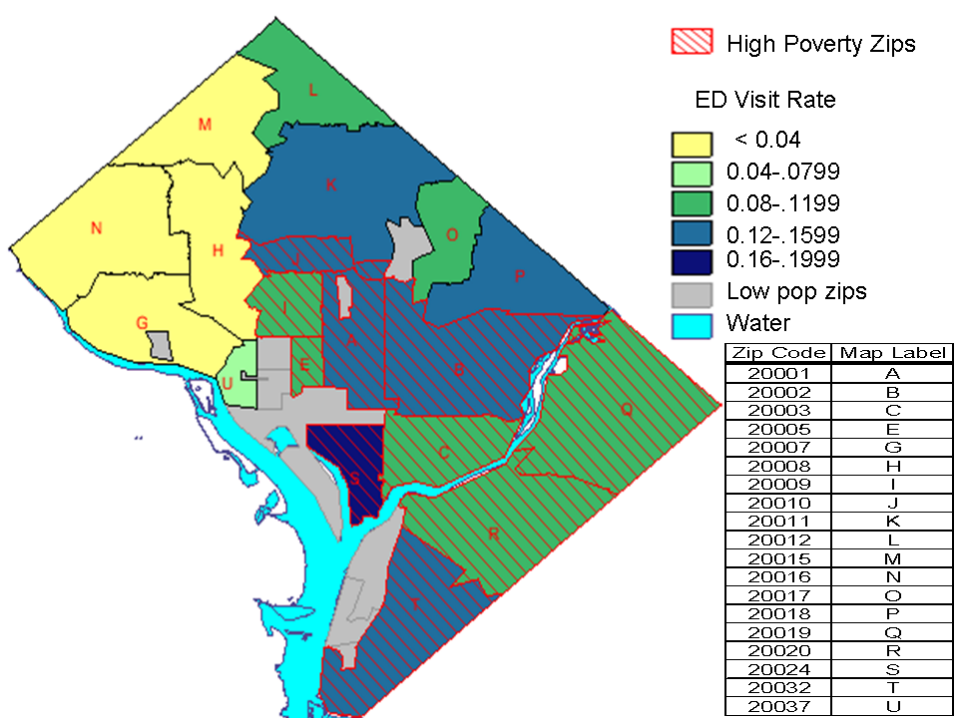
**Asthma-related ED Visit Rates by Zip Code for FY2001-2003**. Asthma-related ED visit rates averaged over fiscal years 2001-2003  (October 2001-September 2004) for ages 1-17 years for zip codes within  DC.  The hatched zip codes are those for which the 2000 US census  determined that 30% or more of the children lived below poverty levels.   The grey zips are those for which there is little or no pediatric  population.

Based on the curves in Fig. 2, we defined "high child poverty" zip codes as those for which the US census data indicated that at least 30% of children under 18 years old lived in households below the poverty level. Based on the 2000 US census, ten DC zip codes met this criterion. The map in Fig. 3 indicates these ten zip codes with cross-hatching. The colors correspond to the asthma-related ED visit rates in the legend. Note that these higher-poverty zip codes tend to have higher ED visit rates than other zip codes.

It is important to emphasize the contextual and ecologic nature of this study. Given the patient's residence zip code, we knew neither which patients within a zip code were in households classified as below the poverty level, nor which patients had sufficient exposure to outdoor air pollutants. With these stipulations, we estimated zip code-level relative risks of acute asthma exacerbation for pediatric residents of high child-poverty zip codes versus those from all DC zip codes. For each relative risk estimate, the numerator was the count of daily ED visits from each residence zip code, and the denominator was a US Census population estimate for the year 2000 for that zip code. Both numerator and denominator were age-group specific (e.g., counts of only 5–12 yr olds used in both numerator and denominator). Table 4 presents these relative risk estimates with upper and lower 95 % confidence limits showing that all estimates are statistically significant. These confidence intervals were calculated using the method of Katz et al [22]. These aggregated calculations support the hypothesis that children of each age group have increased relative risk of requiring an ED visit for asthma exacerbation if their residence is in a high-poverty zip code. The second part of Table 4 presents relative risk estimates in which the numerators were daily counts of only those ED visits that resulted in hospital admission. These tables suggest that, once a child visits an ED for an asthma exacerbation, there is an increased risk of that child needing admission if that child's residence is in a high child poverty zip code. These results are consistent with the curves seen in Fig. 2. Therefore, our results appear to corroborate the hypothesis that, relative to DC children in general, children from high poverty zip codes visit the hospital ED with more severe asthma symptoms, making admission more likely.

**Table 4:** Relative Risk and Conditional Relative Risk for Asthma-related ED Visits and Admissions for High Poverty vs Other Zip Codes

Age Group (yrs)	Outcome	Relative Risk for asthma-related ED visits/admissions for high poverty vs others
1–17	visits	1.45 (1.39 – 1.51)
	admissions	1.75 (1.51 – 2.03)
1–4	visits	1.4 (1.32 – 1.48)
	admissions	1.73 (1.35 – 2.21)
5–12	visits	1.38 (1.3 – 1.47)
	admissions	1.77 (1.42 – 2.2)
13–17	visits	1.71 (1.54 – 1.9)
	admissions	1.73 (1.19 – 2.52)

Age Group (yrs)	Given an asthma-related ED visit, the Relative Risk of asthma- related ED visits being admitted:: high poverty vs others	

1–17	1.2 (1.05 – 1.37)	
1–4	1.23 (1 – 1.52)	
5–12	1.18 (0.98 – 1.43)	
13–17	1.17 (0.85 – 1.62)	

The numbers in parenthesis represent the lower and upper 95 % confidence limits for the percent change in visits and admissions.

## Discussion

We performed Poisson regression analyses of time series of daily counts of pediatric asthma-related hospital ED visits and admissions to seek associations with environmental risk factors. The dataset covered the period from October 1, 2001, through September 30, 2004, and included only DC residents. Separate analyses were done to analyze environmental effects on more serious cases by limiting the subset of counts to those for which the visits resulted in hospital admissions. The risk factors included in the analysis were daily concentrations of ozone, PM2.5, and other environmental factors such as pollen.

During the study period, we found no statistically significant effect of PM2.5 on pediatric asthma-related ED visits or admissions. This result is likely related to the relatively low levels of PM2.5 during our study period (daily PM2.5 never reached Code Red levels). While other studies have shown significant associations between particulates and pediatric ED asthma admissions [23], Fusco et al [24] also found no significant relationship between pediatric asthma and particulates. These different results may be related to different particulate size distributions and concentrations among different locations, different types of sources at those locations (e.g., smokestack industries, diesel vs. gasoline vehicular traffic), and different periods of time.

The 8-hour daily maximum ozone concentrations reached Code Red levels on only five days during the study period. We found some significant associations of ozone and tree pollen on counts of ED visits and admissions for the 1–17 yrs age group, and the largest associations were found on the 5–12 yrs age group. We also calculated significant but smaller associations of ED visits with grass pollen and temperature. Significant ozone associations were seen in the 5–12 yrs age group even when the daily ED visit data were lagged by up to 4 days from the ozone data. Interestingly, regression using grass pollen data did not show a significant same-day effect, but for daily ED visits, this effect became significant for a one-day lag and became stronger as the lag increased to three days before dropping to non-significant levels after three days. Tobias et al [25] noted similar lags of 2 to 3 days, depending on the type of pollen.

The most significant effects of ozone on asthma-related ED visits and admissions were found in the 5–12 yrs age group. Compared to adults, children have a higher alveolar ventilation relative to body mass, as well as a higher peripheral airways resistance, resulting in children having a greater risk of adverse ventilatory effects. As children grow, respiratory rate decreases from about 22 breaths per minute at age 4 to around 14 breaths per minute by age 16 years [26], thereby approaching normal rates for adults. Because the 5–12 yrs age group represents school-age children, it is plausible that this includes a significant number of children who spend a larger amount of time outside than the 1–4 yrs age group. If so, then the 5–12 year old children would be at greater risk for effects of outdoor pollutants. Significant associations found in 5–12 yrs age group for tree and weed pollen concentrations also suggest that this age group may be at greater risk for outdoor exposures triggering reactive airway conditions due to greater time spent outdoors. Ziska et al. [27] observed that ragweed plants in urban locations, where carbon dioxide levels are high, produced more pollen than ragweed plants in rural areas where amounts of carbon dioxide were low. Our result that weed pollen was significantly associated with ED admissions may be related to the fact that ragweed is the primary component of weed pollen and is known to produce huge amounts of pollen that are considered among the most highly allergenic of all pollen.

When averaged over the 36 months of this study, the annual rates of asthma-related ED visits (visits divided by the year 2000 DC census for that age group) were 0.070, 0.033, 0.022, and 0.029 for the age groups 1–4 yrs, 5–12 yrs, 13–17 yrs, and 1–17 yrs, respectively. It is noteworthy that the most significant associations of asthma exacerbations with outdoor environmental factors were not seen in the age group with the highest rate of asthma-related ED visits, the 1–4 yr olds. This may be related to less time spent outdoors and to the clinical difficulty in accurately diagnosing asthma in this age group. Because of the developmental factors mentioned above, children of ages 1–4 yrs may experience significant wheezing and shortness of breath due to allergens or other factors, but these effects often wane as the child develops. Therefore, there is some debate whether these children should have been diagnosed with asthma as opposed to a more temporary reactive airway condition. It is thus possible that diagnoses of asthma in this age group may not represent its true incidence.

An even more interesting finding resulted when we compared pediatric populations from zip codes in which more than 30% of the children lived in households with income classified as below the poverty level (defined as "high child poverty zip codes") with all other zip codes within DC. Fig. 2 reveals a significant logarithmic relationship between the percentage of children living in below-poverty-level households and both asthma-related ED visits and admissions, with each symbol representing a zip code. This figure also shows that the ratio of ED admission to visit rates tends to increase as the percent zip code population below poverty increases. At and above about 30%, the rate of increase in both asthma-related ED visit and admission rates levels off. Increased rates for both asthma-related ED visits and admissions were seen in residents of high child poverty zip codes compared with other zip codes. Table 4 suggests a real increased relative risk of acute asthma exacerbations that require hospital admission among the pediatric residents of high-poverty zip codes. Possible explanations for this observation include postponing primary care until symptoms become severe, discrepancies in outdoor air exposure (e.g., lack of air conditioning), discrepancies in indoor air quality [28], and discrepancies in health care access and availability [29-31]. For example, Levy et al [29] found that only 36% of asthmatic children in three public housing developments in Boston had been prescribed any daily controller medication.

We summarize the limitations of this study. The available data restrict it to a contextual study involving measured outdoor risk factors for a population with a spatial resolution no finer than the zip code level. Krieger et al [32] have shown that inhomogeneities in socio-economic status within a zip code may have statistical impacts that are significant for some health outcomes, such as cancer incidence, and not for others, such as cancer mortality. Our asthma data did not include census tract information. Depending on the homogeneity of DC zip codes for socio-economic status, analysis at the census tract level may be more informative that at the zip code level to which this study was constrained. Another limitation was that the period of our study was only 36 months, while datasets in some similar studies cover much longer time intervals. The spatial analysis of environmental effects was limited in resolution because ozone and PM2.5 data were available from only three stations within DC during the study period. The fact that there were only five Code Red ozone days and no Code Red PM2.5 days during this study suggests limited opportunities to observe relationships between these pollutants and asthma-related ED visits, although asthma-related ED visits may also be associated with conditions below Code Red levels. Despite these limitations, the results of this study, corroborative of similar studies noted above, may be used for hypotheses and designs of more comprehensive, individual-based epidemiological studies.

## Conclusion

We conclude that asthmatic exacerbations, as measured by hospital ED visits and admissions, have significant short-term associations with measured ozone concentrations, especially among 5–12 year olds. The finding that the most significant associations were not seen in the age group with the highest number of asthma-related ED visits, the 1–4 yr olds, may be a combined result of this youngest age group's relatively reduced exposure to outdoor air, especially on high-risk days, and the clinical difficulty in accurately diagnosing asthma in this group. We also found an interesting logarithmic relationship between asthma-related ED visit and admission rates and the percentage of children living below the poverty level within a zip code (Fig. 2). Together, the curves in Fig. 2 suggest that overuse of ED facilities does not explain the increased visits by asthmatic children in high-poverty zip codes and that there appears to be a real increased relative risk of acute asthma exacerbations that require hospital admission among these children. Therefore, the results observed among age and poverty groups above may suggest designs for future studies on efforts to offer health education and services such as asthma maintenance therapy to families in high poverty areas.

## Competing interests

The author(s) declare that they have no competing interests.

## Authors' contributions

SMB participated in the study design and analyses, drafted the manuscript, and provided support as a meteorologist and physician. HSB designed and developed the statistical analyses. RSH coordinated implementation of the study and contributed her expertise in respiratory therapy and public health issues such as socioeconomic status. NRT participated in the spatial analysis and provided geographic information systems analyses. LDS acquired federal funding and collaborated in the study conception, design, and implementation, as well as provided support for understanding the ED data. JODC collaborated in the study conception and implementation. KD provided support for study design and relevant public health issues including socioeconomic status. DHL collaborated in the study conception and design. All authors read and approved the final manuscript.
